# Ppp1r1b-lncRNA inhibits PRC2 at myogenic regulatory genes to promote cardiac and skeletal muscle development in mouse and human

**DOI:** 10.1261/rna.073692.119

**Published:** 2020-04

**Authors:** Xuedong Kang, Yan Zhao, Glen Van Arsdell, Stanley F. Nelson, Marlin Touma

**Affiliations:** 1 Department of Pediatrics, David Geffen School of Medicine, University of California Los Angeles, Los Angeles, California 90095, USA; 2 Neonatal/Congenital Heart Laboratory, Cardiovascular Research Laboratory, University of California Los Angeles, Los Angeles, California 90095, USA; 3 Department of Cardiothoracic Surgery, David Geffen School of Medicine, University of California Los Angeles, Los Angeles, California 90095, USA; 4 Department of Neurology, David Geffen School of Medicine, University of California Los Angeles, Los Angeles, California 90095, USA; 5 Department of Human Genetics, Institute of Precision Health, David Geffen School of Medicine, University of California Los Angeles, Los Angeles, California 90095, USA; 6 Institute of Precision Health, David Geffen School of Medicine, University of California Los Angeles, Los Angeles, California 90095, USA; 7 The Molecular Biology Institute, David Geffen School of Medicine, University of California Los Angeles, Los Angeles, California 90095, USA; 8 Children's Discovery and Innovation Institute, Department of Pediatrics, David Geffen School of Medicine, University of California Los Angeles, Los Angeles, California 90095, USA; 9 Eli and Edythe Broad Stem Cell Institute, David Geffen School of Medicine, University of California Los Angeles, Los Angeles, California 90095, USA

**Keywords:** long noncoding RNA, H3K27me3, epigenome, myogenesis

## Abstract

Long noncoding RNAs (lncRNAs) have emerged as critical epigenetic regulators and play important roles in cardiac development and congenital heart disease. In a previous study, we identified a novel lncRNA, Ppp1r1b, with expression highly correlated with myogenesis. However, the molecular mechanism that underlies Ppp1r1b-lncRNA function in myogenic regulation is unknown. By silencing Ppp1r1b-lncRNA, mouse C2C12 and human skeletal myoblasts failed to develop fully differentiated myotubes. Myogenic differentiation was also impaired in PPP1R1B-lncRNA deficient human-induced pluripotent stem cell-derived cardiomyocytes (hiPSCs-CMs). The expression of myogenic transcription factors, including MyoD, Myogenin, and Tbx5, as well as sarcomere proteins, was significantly suppressed in Ppp1r1b-lncRNA inhibited myoblast cells and neonatal mouse heart. Histone modification analysis revealed increased H3K27 tri-methylation at *MyoD1* and *Myogenin* promoters in GapmeR treated C2C12 cells. Furthermore, Ppp1r1b-lncRNA was found to bind to Ezh2, and chromatin isolation by RNA purification (ChIRP) assay revealed enriched interaction of Ppp1r1b-lncRNA with *Myod1* and *Tbx5* promoters, suggesting that Ppp1r1b-lncRNA induces transcription of myogenic transcription factors by interacting with the polycomb repressive complex 2 (PRC2) at the chromatin interface. Correspondingly, the silencing of Ppp1r1b-lncRNA increased EZH2 binding at promoter regions of myogenic transcription factors. Therefore, our results suggest that Ppp1r1b-lncRNA promotes myogenic differentiation through competing for PRC2 binding with chromatin of myogenic master regulators during heart and skeletal muscle development.

## INTRODUCTION

Long noncoding RNAs (lncRNAs), which comprise the bulk of the noncoding genome, have been proven to play important roles in maintaining cardiovascular system homeostasis (Ounzain et al. 2013; Touma et al. 2016). Therefore, exploring the mechanisms that underlie their function in heart development will further deepen our understanding of heart development and provide new insight for promoting translational applications.

Skeletal and cardiac muscle both arise from myogenic mesodermal lineages and share many characteristics. It was reported that the expression patterns of cardiac and skeletal muscle transcription factors and fast-skeletal myosin heavy chain (sk-fMHC) within ventricular myocardium and skeletal muscle were similar at embryonic day (ED) 20, and the expression patterns became cardiac or skeletal muscle specific only during postnatal development (Clause et al. 2012). Some types of muscular dystrophies also associate with cardiomyopathy and chronic cardiac diseases (Muntoni 2003). The overlapping pattern of muscle-specific gene expression in cardiac and skeletal muscle and the common origin of cardiac and skeletal muscle pathologies suggest the existence of a common underlying regulatory scheme for the control of muscle genes expression, especially in their immature phase (Hassan et al. 2014).

Several myogenic transcription factors have been identified. Among them, the basic helix–loop–helix (bHLH) family (MYOD1, MYF5, and MYOG) were discovered to be key regulators of muscle development (Hernández-Hernández et al. 2017). Likewise, heart development is governed by a core set of transcription factors (NKX2.5, MEF2, GATA, TBX, and Hand) (Garry and Olson 2006). However, these known core set of transcription factors mainly controls the expansion of chamber myocardium but not the initial differentiation and commitment to cardiomyocytes (Garry and Olson 2006; Olson 2006). The precise molecular mechanisms of the initial differentiation are not entirely clear in heart development. Therefore, detailed understanding of the mechanisms of the initial differentiation is necessary, especially for cardiomyocyte regeneration.

Epigenetic modifications at the transcription factor binding sites play critical roles in the regulation of gene expression, including the myogenic differentiation genes (Kouzarides 2007). In embryonic stem cells or induced pluripotent stem cells (ESCs/iPSCs), H3K27me3 functions as an “epigenetic barrier” against ESC/iPSC differentiation. In the meantime, H3K27 demethylation facilitates MyoD1-mediated myogenic differentiation (Aloia et al. 2013; Adam and Fuchs 2016). During direct cardiac reprogramming, increased level of H3K27me3 at the promoter sites of cardiac transcription factors, such as Mef2c, Gata4, and Tbx5, corresponds to decrease of mRNA expression of cardiac genes (Liu et al. 2016).

LncRNAs may carry out both gene inhibition and gene activation through a range of diverse mechanisms. Targeted modulation of epigenetic modifiers, such as the Polycomb repressive complex (PRC) subunits, by lncRNAs has been suggested as a general mechanism for trans-acting lncRNA in gene regulation (Prasanth and Spector 2007). PRC-mediated gene silencing is mainly dependent on the EZH2 dependent H3K27 tri-methylation (Schuettengruber and Cavalli 2009). LncRNAs can directly modulate EZH2 activity or recruit EZH2 to the promoter region of genes to achieve targeted gene repression (Su et al. 2018). Moreover, lncRNAs can also serve as EZH2 effectors or regulators (Su et al. 2018). For the inhibition of chromatin methylation, a model was suggested that the interactions of PRC2 with lncRNAs and chromatin are mutually antagonistic (Wang et al. 2017) and lncRNAs inhibit PRC2 methyltransferase activity by blocking PRC2 binding to targeted chromatin (Beltran et al. 2016).

In this report, we investigated the expression patterns of Ppp1r1b-lncRNA during myoblast differentiation, assessed the effect of down-regulation of Ppp1r1b-lncRNA on myogenesis in vitro and in vivo, and examined its impact on H3K27 methylation status at promoter regions of myogenic transcription factors, including MyoD, Myogenin, and Tbx5. We demonstrated interaction between Ppp1r1b-lncRNA, with Ezh2, and targeted promoters of myogenic transcription factors. These findings revealed that Ppp1r1b-lncRNA regulates myocyte differentiation by negatively modulating H3K27me3 of myogenic transcription factor genes via competing for PRC2 binding with chromatin.

## RESULTS

### Ppp1r1b-lncRNA is overexpressed in differentiated C2C12 cells in association with myogenesis

**FIGURE 1.**
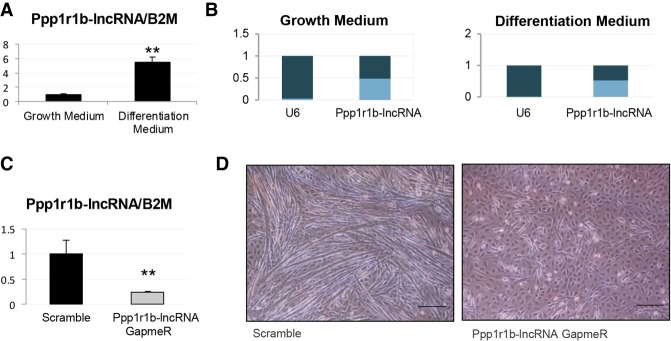
Ppp1r1b-lncRNA is induced during myogenesis and regulates C2C12 myoblast differentiation. (*A*,*C*) Quantitative real-time PCR analysis of Ppp1r1b-lncRNA expression after 2 d of differentiation (*A*) and GapmeR treatment (*C*). (*B*) Subcellular localization of Ppp1r1b-lncRNA in myoblasts. U6 RNA was used as a nuclear location control. (*D*) Light microscope images depict the morphology of C2C12 cells after 2 d of differentiation and Ppp1r1b-GapmeR, or Scramble, treatment. Scale bar, 200 µm. *N* = 4 biological replicates per condition; error bars, standard error of the mean; (**) *P* < 0.01.

To determine the effects of Ppp1r1b-lncRNA on myogenesis, C2C12, an immortalized mouse myoblast cell line, was used. We cultured C2C12 cells in high serum conditions for rapid proliferation and low serum conditions for differentiation. The expression of Ppp1r1b-lncRNA in differentiated C2C12 cells was significantly higher than that in the nondifferentiated cells (Fig. 1A). Subcellular localization of Ppp1r1b-lncRNA was characterized after subcellular fractionation of C2C12 cells. Ppp1r1b-lncRNA was equivalently distributed between nucleus and cytoplasm in both nondifferentiated and differentiated cells (Fig. 1B).

In order to elucidate the functional role of Ppp1r1b-lncRNA, we treated C2C12 cells cultured in differentiation media with 50 nM antisense Ppp1r1b-lncRNA GapmeR for 48 h. The GapmeR treatment decreased Ppp1r1b-lncRNA expression by more than 70% in differentiated C2C12 cells (Fig. 1C). Morphology of cells was visualized with a light microscope (Fig. 1D). A significant lower degree of differentiation was observed in the GapmeR treated cells that remained in a quiescent mono-nucleated status and did not differentiate into myotubes. These results indicate that Ppp1r1b-lncRNA expression is required for normal myogenesis.

### Human PPP1R1B-lncRNA exhibits similar roles in myogenesis of human myoblast

LncRNAs are known to be poorly conserved across species. Furthermore, lncRNAs are less conserved at the primary nucleotide sequences compared with protein coding genes (Paralkar et al. 2014). It was reported that the repeat-containing regions in Xist lncRNA are generally unstructured and are functionally bound by protein cofactors (Smola et al. 2016). However, using comparative sequence, structural, and functional analyses, Karner et al. (2019) demonstrated that the functional conservation of lncRNA is independent from sequence and structural divergence (Karner et al. 2019). Therefore, determining functionally conserved and specific Ppp1r1b-lncRNA in human is essential. The human PPP1R1B locus has three noncoding transcripts. By comparing PPP1R1B-lncRNA transcripts between mouse and human, we identified a potential human ortholog of Ppp1r1b-lncRNA. However, unlike the mouse Ppp1r1b-lncRNA, in which no open reading frame (ORF) was detected, the human PPP1R1B-lncRNA contained several short ORFs that can potentially produce small peptides. To examine the coding potential, we cloned the full-length mouse and human lncRNAs and carried out an in vitro expression and translation assay (Materials and Methods; Supplemental Fig. 1). Consistent with lncRNAs properties, no peptide was produced confirming the lack of a functional ORF in both mouse and human orthologs.

**FIGURE 2.**
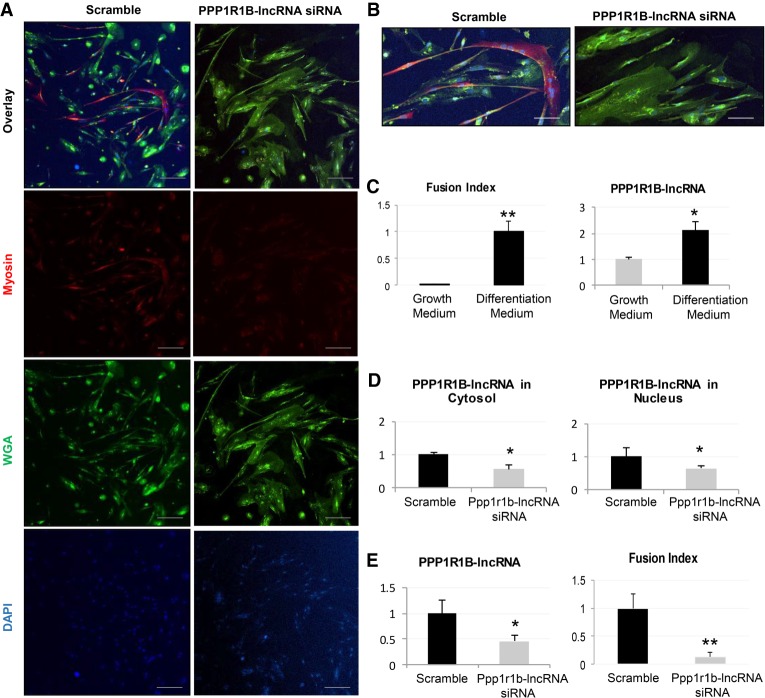
Human PP1R1B-lncRNA promotes differentiation of human skeletal myoblast. (*A*) Fluorescent microscope images depict inhibition of myogenesis of human skeletal myoblast (myosin positive cells) after PPP1R1B-lncRNA siRNA treatment. Scale bar, 100 µm. (*B*) Higher magnification images of cells presented in *A*. (*C*) Real-time PCR analysis and fusion index analysis of human myoblast before and after differentiation (625 nuclei counted for growth medium; 571 nuclei counted for differentiation medium). (*D*) RNA fractionation analysis of PPP1R1B-lncRNA down-regulated by siRNA. (*E*) Real-time PCR analysis and fusion index analysis of human myoblast after PPP1R1B-lncRNA siRNA treatment in differentiation medium (737 nuclei counted for Scramble; 760 nuclei counted for siRNA). *N* = 4 biological replicates per condition; error bars, standard error of the mean; (*) *P* < 0.05; (**) *P* < 0.01.

Like in mice, the human PPP1R1B-lncRNA was also induced during differentiation (Fig. 2A–C). To examine whether the human ortholog carries a similar function, we first used siRNAs directed against human PPP1R1B-lncRNA in a human myoblast cell line and achieved efficient suppression in the nuclear and cytosolic compartments (Fig. 2D). Myotube differentiation was also impaired, as demonstrated by decreased myosin protein expression compared to control cells (Fig. 2A,B) and reduced fusion index (Fig. 2E), defined as the number of nuclei in myosin heavy chain (DSHB) positive cells divided by the total number of nuclei per surface area. These results indicate that PPP1R1B-lncRNA has a conserved function in human myogenesis.

### Human PPP1R1B-lncRNA exhibited similar roles in myogenesis of human cardiomyocytes

**FIGURE 3.**
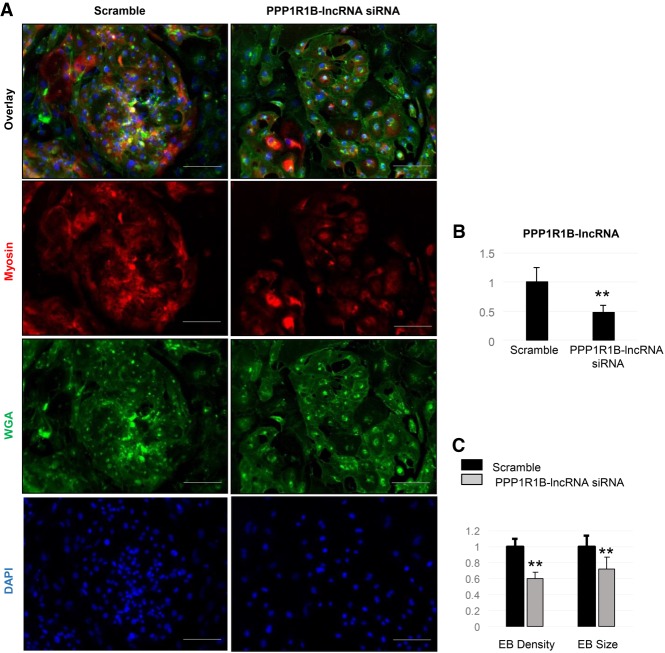
Human PPP1R1B-lncRNA promotes differentiation of human iPSC-derived cardiomyocyte (hiPSC-CM). (*A*) Microscope images depict the differentiation efficiency of human hiPSC-CM (Myosin positive cells) after PPP1R1B-lncRNA siRNA, or Scramble, treatment. (WGA) Wheat germ agglutinin. Scale bar, 100 µm. (*B*) Quantitative real-time PCR analysis of PPP1R1B-lncRNA expression after siRNA treatment. (*C*) Quantitative analysis depicts differentiation efficiency of hiPSC-CMs after siRNA treatment. (EB) Embryonic bodies. *N* = 4 biological replicates per condition; error bars, standard error of the mean; (*) *P* < 0.05; (**) *P* < 0.01.

LncRNAs are known to have tissue specificity. To examine whether the human specific PPP1R1B-lncRNA affect myogenesis during cardiac muscle development, we used a human-induced pluripotent stem cell-derived cardiomyocyte (hiPSC-CM) cell line. By silencing the PPP1R1B-lncRNA, myogenic differentiation of cardiomyocytes was impaired. The iPSC-CMs also exhibited significantly less myosin expression and myofibrillar bundles following PPP1R1B-lncRNA siRNA treatment (Fig. 3A,B). Furthermore, PPP1R1B-lncRNA knockdown led to significant diminution of the sizes of the differentiation aggregates, which are known as embryonic bodies (EBs). The EB density, defined as the total number of nuclei in EB divided by the surface EB area, was also decreased (Fig. 3C). These results further confirmed a conserved function for the human PPP1R1B-lncRNA in promoting myogenesis in heart.

### Ppp1r1b-lncRNA positively regulates muscle-specific transcription factors and muscle structure genes

To further characterize the function of Ppp1r1b-lncRNA in myogenesis, we examined the impact of its loss on mRNA expression of the muscle-specific transcription factors and muscle structure genes. It was known that the differentiation of skeletal muscle cells is governed by a group of four myogenic regulatory factors (MRFs) including MyoD, Myf5, Myogenin and MRF4 (Buckingham and Rigby 2014). MyoD is generally believed to act as a fate determination gene, while myogenin is essential for the terminal differentiation.

**FIGURE 4.**
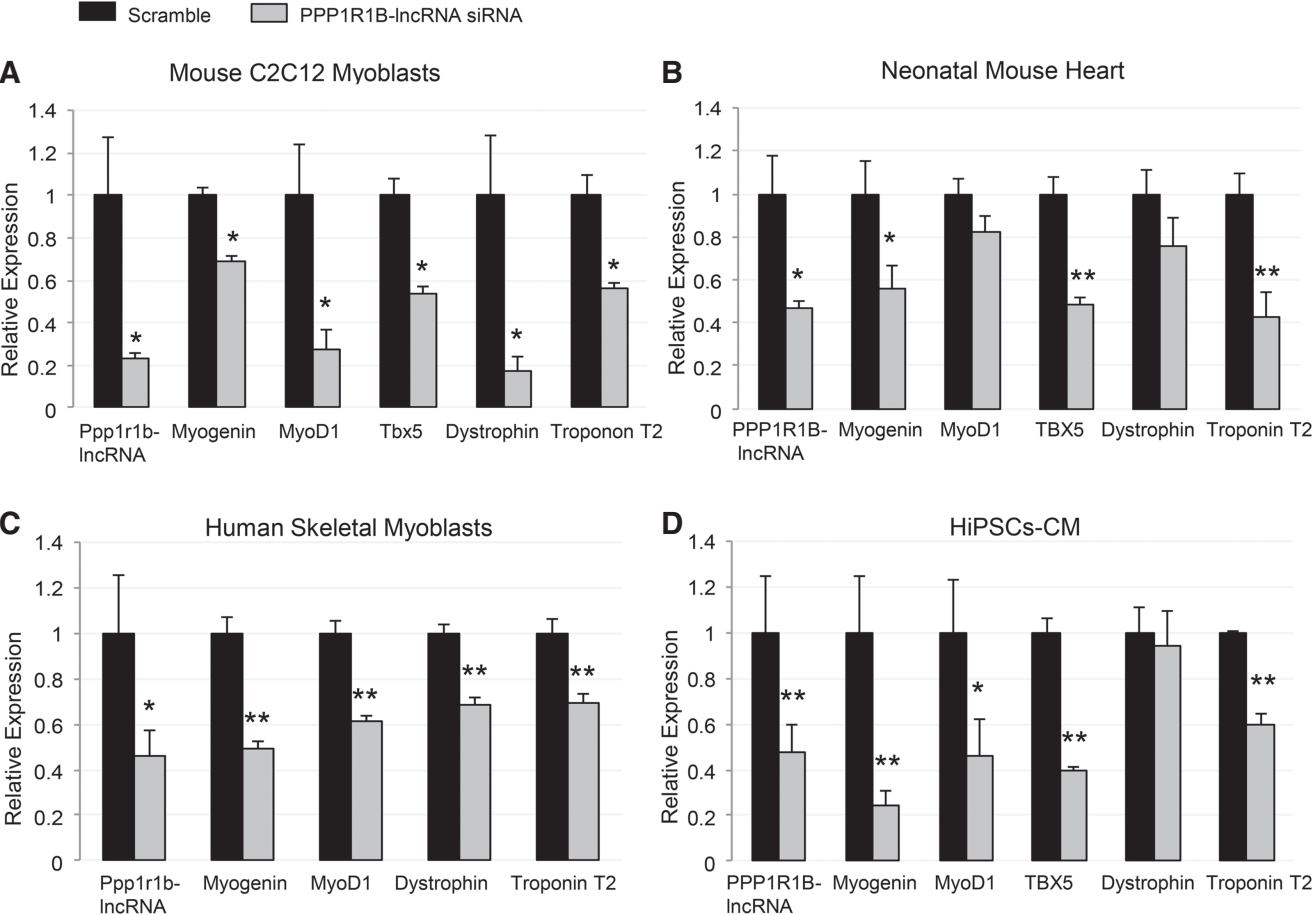
Ppp1r1b-ncRNA regulates expression of myogenic regulatory factors and sarcomere structural genes. Real-time PCR analysis of myogenic regulatory factors and sarcomeric structural genes after Ppp1r1b-lncRNA silencing in Mouse C2C12 myoblasts (*A*), mouse heart tissue (*B*), human skeletal myoplasts (*C*), and human-induced pluripotent stem cell-derived cardiomyocytes (hiPSCs-CM) (*D*). *N* = 4 biological replicates per condition. Error bars, standard error of the mean. (*) *P* < 0.05; (**) *P* < 0.01.

Our data revealed that mRNA levels of Myogenin, MyoD1, Tbx5, Dystrophin and Troponin T2, were significantly lower in the GapmeR treated C2C12 cells compared with the untreated group (Fig. 4A). Likewise, siRNA treated human myoblasts showed a significant decrease in mRNA expression of the same set of myogenic transcription factors and muscle structural genes (Fig. 4B). Furthermore, GapmeR mediated Ppp1r1p-lncRNA silencing in neonatal mouse heart tissue revealed a similar trend (Fig. 4C). Moreover, siRNA treated hiPSC-CM also showed significant decrease in mRNA expression of muscle-specific transcription factors, including TBX5 and muscle structure genes (Fig. 4D). Taken together, the above findings suggest that Ppp1r1b-lncRNA modulates myogenesis by positively regulating myogenic transcription factors and structural genes of striated muscles development in both mouse and human myoblasts, cardiac progenitors, as well as neonatal mouse heart.

### Ppp1r1b-lncRNA negatively modulates histone methylation on the promoter of muscle-specific transcription factors

**FIGURE 5.**
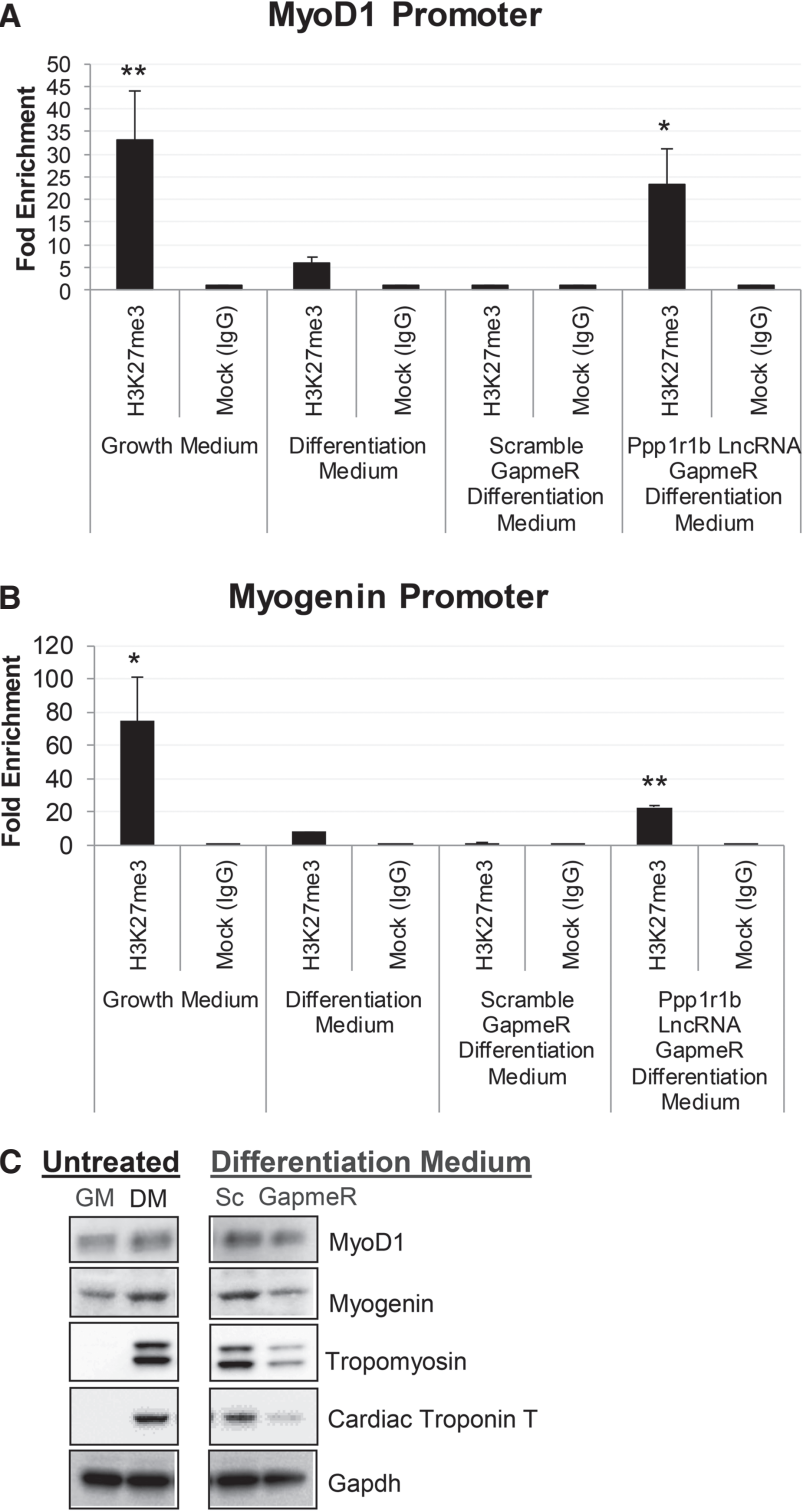
Ppp1r1b-lncRNA inhibits Histone 3 methylation at the promoter regions of myogenic regulatory factors. C2C12 myoblasts were cultured in growth medium (GM) or differentiation medium (DM) and treated by scramble GapmeR or Ppp1r1b-lncRNA GapmeR. After treatment, cells were subjected to ChIP analysis using anti H3K27me3 antibodies. The qPCR data are presented as fold enrichment to the background, indicating the abundance of histone modifications at the promoter regions of the myogenic genes. (*A*) H3K27me3 level on MyoD1 promoter. (*B*) H3K27me3 level on Myogenin promoter. (*C*) Western blot analysis of myogenic regulatory factors and sarcomeric structural proteins from C2C12. Gapdh was used as a loading control. (GM) Growth medium, (DM) differentiation medium. (*) *P* < 0.05; (**) *P* < 0.01.

It was reported that lncRNAs can precisely regulate skeletal muscle proliferation and differentiation by regulating H3K27me3 levels (Gan et al. 2019). To examine the epigenetic effects of Ppp1r1b-lncRNA on myogenesis associated gene expression, we performed ChIP-PCR analysis for H3K27me3 in C2C12 cells. As shown in Figure 5A,B, the H3K27me3 levels on *MyoD1* and *Myogenin* promoters were decreased in the differentiated myotubes compared to the undifferentiated myoblasts. After Ppp1r1b-lncRNA GapmeR treatment, the H3K27me3 levels were increased. These findings are consistent with the suppressed expression of these transcription regulators (Fig. 5C) and impaired differentiation after GapmeR treatment. From the above results, we deduced that Ppp1r1b-lncRNA positively regulates the expression of myogenic transcription factors by negatively modulating H3K27me3 levels on their promoters.

### Ppp1r1b-lncRNA interacts with PRC2 core member, EZH2

**FIGURE 6.**
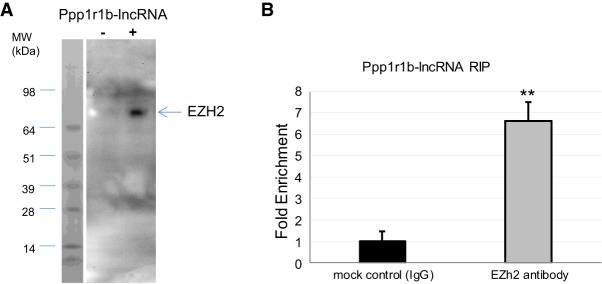
Ppp1r1b-lncRNA binds EZH2. (*A*) RNA pull-down assays were carried out by incubating cell lysate from neonatal mouse heart tissue with biotinylated in vitro transcribed Ppp1r1b-lncRNA. Subsequent western blotting analysis revealed EZH2 in the isolated RNA-protein complex. (*B*) RNA immunoprecipitation (RIP) demonstrated that Ppp1r1b-lncRNA was immunoprecipitated together with EZH2 in neonatal mouse heart tissue. LncRNA was detected using real-time PCR. (*) *P* < 0.05.

Polycomb repressive complex 2 (PRC2) possesses histone methyltransferase (HMT) activity with specificity for Lys 9 (K9) and Lys 27 (K27) of histone H3 (Kuzmichev et al. 2002). EZH2 is the functional enzymatic component of PRC2. To investigate the potential interaction between Ppp1r1b-lncRNA and PRC2, we performed an RNA pulldown assay using neonatal mouse heart tissue and demonstrated that Ezh2 protein co-precipitated with biotinylated Ppp1r1b-lncRNA (Fig. 6A). We next performed an RNA immunoprecipitation (RIP) assay using Ezh2 antibody, and detected an immune-complex of Ezh2 isolated from chromatin fractions with enriched Ppp1r1b-lncRNA (Fig. 6B). These results suggest that Ppp1r1b-lncRNA interacts directly with Ezh2 complex in the myocytes.

### Interaction of Ppp1r1b-lncRNA with *MyoD1* and *TBX5* promoters

**FIGURE 7.**
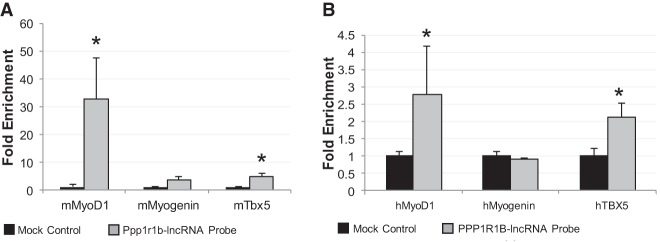
Ppp1r1b-lncRNA binds to MyoD1, Myogenin and Tbx5 promoter region. Chromatin isolation by RNA purification (ChIRP) assays were carried out by incubating cell lysate from mouse neonatal heart tissue and human infantile heart tissue with biotinylated in vitro synthesized Ppp1r1b-lncRNA probes. Promoter DNAs were detected by real-time PCR. (*A*) Interactions of Ppp1r1b-lncRNA with MyoD1, Myogenin, and Tbx5 promoter DNA in mouse. (*B*) Interactions of Ppp1r1b-lncRNA with MyoD1, Myogenin, and Tbx5 promoter DNA in human. (*) *P* < 0.05.

**FIGURE 8.**
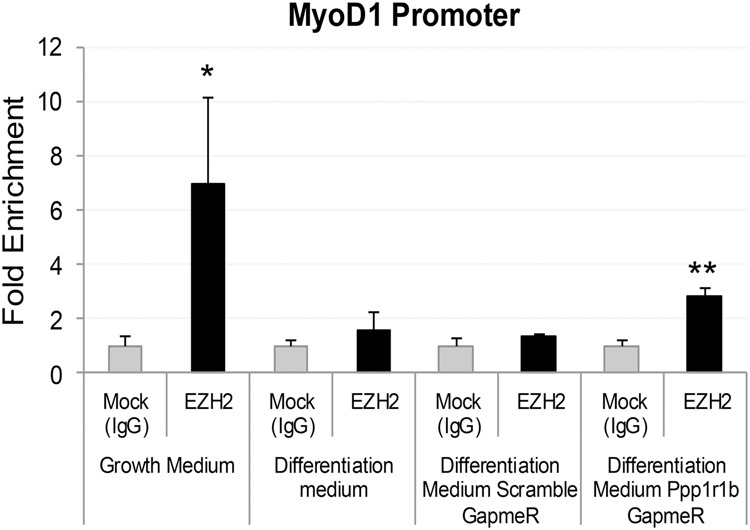
Ppp1r1b-lncRNA Inhibits EZH2 Binding at the MyoD1 promoter region. C2C12 myoblasts were cultured in growth and differentiation medium (DM) treated with scramble GapmeR and Ppp1r1b-lncRNA GapmeR. Treated C2C12 cells were subjected to ChIP analysis using anti-EZH2 antibodies. The qPCR data are presented as fold enrichment to the background, indicating the EZH2 abundance at the MyoD1 promoter region. (*) *P* < 0.05; (**) *P* < 0.01.

The association of PRC2 with chromatin is thought to be regulated by interaction with RNA. We showed that higher Ppp1r1b-lncRNA expression was correlated with lower H3K27me3 levels at the promoters of myogenic transcription factors (Fig. 5). We conducted chromatin isolation by RNA purification (ChIRP) using mouse specific and human specific Ppp1r1b-lncRNA probes, respectively. Promoter DNA of several myogenic regulators, including Myogenin, MyoD1 and Tbx5*,* was analyzed from the pulled RNA: DNA interactome (Fig. 7). Among them, *MyoD1* and *Tbx5* promoter DNA were significantly enriched in the Ppp1r1b-lncRNA probe isolated genomic DNA compared to a negative control probe (Fig. 7). However, *Myogenin* promoter DNA was not enriched, suggesting that MyoD1 is the most important myogenic factor regulated by Ppp1r1b-lncRNA. To further determine how Ppp1r1b-lncRNA inhibits histone methylation and correspondingly increases MyoD1 expression, we conducted a ChIP assay using EZH2 antibody, combined with Ppp1r1b-lncRNA silencing. The association of PRC2 with *MyoD1* promoter DNA was negatively regulated by Ppp1r1b-lncRNA (Fig. 8). These results indicate that Ppp1r1b-lncRNA inhibits H3K27 methylation at *MyoD1* promoter regions by eviction of PRC2.

## DISCUSSION

In this study, we show that Ppp1r1b-lncRNA is necessary for normal myogenesis. By knocking down Ppp1r1b-lncRNA, the expressions of myogenic transcription factors were significantly reduced. Although the lncRNAs are known to have species specificity and tissue specificity, Ppp1r1b-lncRNA appears to be a conserved transcriptional regulator in both human and mouse, and in both cardiac and skeletal muscle. It has been proven that developing skeletal muscle shares similar genetic network and common cell lineage with cardiac muscle during development. Du et al. (2008) found that noncardiac MHCs play a major role during the early sarcomere structure assembly in cardiomyocytes. Recently, it was revealed that cardiac Troponin I and T are also expressed in developing skeletal muscle (Sehnert et al. 2002; Clause et al. 2012). Thus, we can hypothesize that Ppp1r1b-lncRNA may modulate the initial differentiation via a common molecular mechanism, contributing to similar molecular features in the heart and skeletal muscle.

H3K27me3 at promoter sites is a marker of transcriptional repression. It was reported that removal of this repressive marker is essential for the induction of cardiac reprogramming (Dal-Pra et al. 2017). The demethylation of H3K27me3 facilitates MyoD1-mediated myogenic differentiation (Akiyama et al. 2017). By examining the levels of trimethylated histone H3 of lysine 27 from GapmeR treated C2C12 cell, our results showed that the silencing of Ppp1r1b-lncRNA increased the enrichment of H3K27me3 to predifferentiation levels, concurrent with a decrease in the expression of MyoD1 and Myogenin, at both mRNA and protein levels. Therefore, Ppp1r1b-lncRNA functions to promote MyoD1 and Myogenin transcription by negatively modulating the level of H3K27me3. However, the complete scope of Ppp1r1b-lncRNA targeted genes beyond *MyoD1* and *Myogenin* during myogenesis remains to be fully characterized.

The EZH2 protein is the enzymatic component of PRC2 which catalyzes tri-methylation of histone H3 lysine 27. Using RIP, RNA pulldown and ChIRP analyses, we have demonstrated that Ppp1r1b-lncRNA interacts with EZH2, and binds on promoters of *MyoD1* and *TBX5*. These results suggest that Ppp1r1b-lncRNA physically associates with PRC2 complex at promoters of myogenic transcription factors. The inhibition of H3K27me3 by Ppp1r1b-lncRNA could be due to inhibition of PRC2 activity or eviction of PRC2 from chromatin by Ppp1r1b-lncRNA (Yan et al. 2019). It was also proposed that PRC2 recruitment or eviction is determined by the rate at which RNA is released from the locus. At a slow release rate, the majority of RNA remains attached to chromatin and thus PRC2-RNA-chromatin are kept in close proximity. However, at high release rate, most of the RNA is freed from chromatin. Hence, PRC2 is titrated away by RNA from chromatin (Prasanth and Spector 2007). Our results propose that Ppp1r1b-lncRNA precludes PRC2 recruitment to MyoD1 promoter by competing for PRC2 binding.

In our study, we identified Ppp1r1b-lncRNA as a required regulator of myogenesis in striated, skeletal and cardiac muscle cells. It efficiently works on some myogenesis transcription factors such as MyoD1 and Tbx5 to initiate the differentiation of skeletal myoblasts and hiPSCs-derived cardiomyocytes. In skeletal muscle, MyoD has been identified as a master regulator of differentiation (Davis et al. 1987). It was also reported that MyoD depletion leads to a dystrophy-associated cardiomyopathy (Megeney et al. 1999). Some types of muscular dystrophy are also associated with cardiomyopathy (Muntoni 2003). Progression of skeletal muscle damage is a significant contributing factor leading to development of cardiomyopathy (Megeney et al. 1999). Thus, we speculate that together with MyoD, Ppp1r1b-lncRNA may play a role in cardiac diseases progression. Further studies are needed to explore this potential role.

The cardiac transcription factor Tbx5 is a key regulator of heart development (Steimle and Moskowitz 2017). During early cardiac development, Tbx5 appears to act primarily as a transcriptional activator of genes associated with cardiomyocyte maturation and upstream of the morphological signals for septation. Human mutations in TBX5 cause congenital heart disease (CHD) (Baban et al. 2014; Al-Qattan and Abou Al-Shaar 2015), although the underlying mechanism is unknown. It is reported that Tbx5 is regulated through microRNA dependent mechanisms (Wang et al. 2014) and may play a role in adult conduction defects and pathological remodeling in diseased hearts (Torrado et al. 2015). To date, there is no report about interaction between the *Tbx5* promoter and lncRNA. Our results, for the first time, demonstrated that the newly identified Ppp1r1b-lncRNA is involved in Tbx5 regulation during cardiac myogenesis and development, although further studies will be needed to elucidate the role of Ppp1r1b-lncRNA in regulating Tbx5 expression.

Our study has revealed the crucial roles of Ppp1r1b-lncRNA in myogenesis control and potential molecular mechanisms. Heart formation encompasses an orchestrated series of molecular and cellular events, and thus even subtle alterations in this process can lead to serious cardiac disorders. On the other hand, cardiac muscle has limited proliferative capacity and regenerative therapies are highly in demand as a new treatment strategy. Our results may facilitate our understanding of the function of Ppp1r1b-lncRNA and provide us with a promising therapeutic target for congenital heart disease and skeletal myopathy. However, we should acknowledge important limitations: (i) Although several models have demonstrated the molecular mechanisms involved in the lncRNA dependent inhibition of histone methylation (Wang et al. 2017), further studies are needed to elucidate the detailed mechanisms of Ppp1r1b-lncRNA function in the regulation of MyoD1 and Tbx5. In addition, the mechanism by which Ppp1r1b-lncRNA regulates *Myogenin* expression remains to be investigated. (ii) While our results support a role for the Ppp1r1b-lncRNA during early stages of muscle and heart development, our in vivo studies are limited to temporal Ppp1r1b-lncRNA silencing during the neonatal period. A comprehensive in vivo characterization of the functional roles during development in transgenic mouse models is required. (iii) Examining the translational value of Ppp1r1b-lncRNA manipulation requires temporal and tissue specific in vivo delivery of Ppp1r1b-lncRNA in disease models of muscular dystrophy, cardiomyopathy, and congenital heart disease.

## MATERIALS AND METHODS

Extended materials and their sources are available in the Supplemental Information Appendix.

### Cell lines and human heart tissue

C2C12 and Human skeletal muscle myoblast cell lines were obtained from ATCC, and ZenBio. Human iPSC-derived cardiomyocytes (SCVI480-CM) were obtained from Joseph C. Wu MD, PhD at the Stanford Cardiovascular Institute (CVI), Stanford; CA. Cells were cultured according to protocols established at the CVI (Wu et al. 2019). Human heart specimens were obtained from the UCLA-Translational Pathology Core Laboratory (TPCL) Core and the UCLA Congenital Heart Defect-BioCore (Touma et al. 2017). Human studies were conducted in accordance with regulation of the University of California Los Angeles Institutional Review Board.

### Mouse injection and in vivo study

All animal-related experimental protocols were approved by the University of California Los Angeles Animal Care and Use Committee (ACUC). Wild-type C57BL/6 mice were purchased from Charles River laboratory. Neonatal mice were used for Ppp1r1b-lncRNA GapmeR injection in vivo. C57BL/6 neonatal mouse pups were intravenously injected with 1.2 nmol/g body weight GapmeR at P1, P3, and P5. Mice were sacrificed at P7 and heart tissues were collected. Experimental protocols were approved by the University of California Los Angeles Animal Care and Use Committee.

### Cell transfection

Transient transfection of cells with GapmeR (Qiagen) and siRNA (Bioland Scientific) was performed in 12-well plates using Lipofectamine 2000 reagent or RNAiMAX (Life Technologies). Mouse Ppp1r1b-lncRNA GapmeR (50 pmol/well) was transfected into C2C12 cells cultured in differentiation media. Human PPP1R1B-lncRNA siRNA (30 pmol/well) was transfected into human skeletal muscle myoblast and human iPSC-CM cultured in differentiation media.

### Isolation of cytoplasmic and nuclear RNA

Cytoplasmic and nuclear RNA were isolated with the Cytoplasmic and Nuclear RNA Purification Kit (Norgen Biotek) following the manufacturer's manual.

### Real-time PCR analysis

Total RNA was isolated from the cultured cells or tissue using RNeasy Mini Kit (Qiagen). The reverse transcription reaction was performed with 1 µg total RNA and the SuperScript IV First-Strand Synthesis System (Thermo Fisher Scientific), and the real-time PCR reactions were performed by using iTaq Universial SYBR Green Supermix from BioRad. Primers were prepared by Invitrogen. (Supplemental Information Appendix, Table S1). Relative expression value was calculated using the comparative threshold cycle (ΔΔCT) method.

### Western blotting

Cell extracts from cultured cells or tissue were fractionated by SDS-PAGE. The iBlot 2 Dry Blotting System and iBind Western System (Thermo Fisher Scientific) were used to transfer proteins to a membrane for analyzing with antibodies. Antibodies were listed in Supplemental Information Appendix, Table S2.

### Immunofluorescence staining

Cells were fixed in 4% (v/v) formaldehyde in PBS for 20 min. After rinsing with PBS, the cells were incubated with 0.1% TritonX-100 in PBS for 15 min at room temperature and rinsed. After blocking in PBS containing 10% Bovine Serum Albumin for 1 h, the cells were incubated overnight with primary antibody and then appropriate AlexaFluor conjugated secondary antibodies for 1 h. Cell nuclei were eventually counterstained by DAPI. Images were recorded on a Nikon confocal microscope and an echo revolve microscope (San Diego, CA). Fusion index was defined as the number of nuclei in myosin heavy chain (DSHB) positive cells divided by the total number of nuclei. EB density was defined as the total number of nuclei in EB divided by the EB area.

### In vitro expression and translation assay

The full-length mouse and human lncRNA cDNA was cloned into pCMV Sport 6.1 vector, which contains a T7 or a SP6 promoter. The Transend nonradioactive system was used to incorporate biotinylated lysine into nascent proteins. After the in vitro TNT reaction, the reaction was terminated by adding SDS loading buffer and incubation at 95°C for 5 min. After SDS-PAGE and electro blotting, the biotinylated proteins were visualized by binding Streptavidin-HRP and chemiluminesent detection.

### Chromatin immunoprecipitation (ChIP)

Undifferentiated and differentiated C2C12 as well as Ppp1r1b-lncRNA GapmeR treated C2C12 cells were exposed to 1% formaldehyde, and chromatin was fragmented by sonication. Immunoprecipitation was performed using IgG (negative control) or with the primary antibodies for H3K27me3 or EZH2. DNA was purified from the immunoprecipitates using the PCR purification kit (Qiagen). Subsequently, real-time PCR was performed to amplify the promoter region DNA of Myogenin and MyoD1. Primers were prepared by Invitrogen (Supplemental Information Appendix, Table S1). Data were analyzed using fold enrichment method.

### RNA pulldown

The assay was performed with P0 C57BL/6 mouse hearts. T7 RNA polymerase kit (Promega) and Biotin RNA labeling mix (Roche) were used for preparation of biotin-labeled Ppp1r1b-lncRNA. After incubated biotinylated RNA with cell extract at 4°C, the washed Dynabeads M280 Streptavidin (Thermo Fisher Scientific) was added to the pulldown mixture. After 1 h incubation at 4°C for 1 h, the beads were collected by using the magnetic apparatus and washed with the binding buffer. Proteins that were co-precipitated were eluted in the SDS sample buffer and fractionated in 8%–16% gradient SDS-PAGE for western analysis with EZH2 antibody (Supplemental Information Appendix, Table S2).

### RNA immunoprecipitation (RIP)

RNA immunoprecipitation experiments were performed with P1 neonatal mouse heart tissue. Tissue lysate was cross-linked by 1% formaldehyde and lysed by sonication. After EZH2 antibody (Supplemental Information Appendix, Table S2) was added to cell lysate and incubated overnight at 4°C, Protein A/G beads (Thermo Scientific) were added and incubated for 1 h at 4°C with gentle rotation. After washed off unbound material, RNAs bound to immunoprecipitated EZH2 were isolated and qRT–PCR was performed using Ppp1r1b-lncRNA primers (Supplemental Information Appendix, Table S1).

### Chromatin isolation by RNA purification (CHIRP)

The Magna CHIRP RNA interactome kit (EMD Millipore Corp.) was used. Assays were performed per manufacturer's protocol. Capture probes were designed to target Ppp1r1b-lncRNA and prepared at 50 µM total oligo concentration. P1 mouse heart tissue and human fetal heart tissue were cross-linked in 1% glutaraldehyde/PBS and sonicated to shear DNA. Probes were added to cell lysate and incubated overnight for hybridization. After hybridization, Streptavidin magnetic beads were added and incubated for an additional 30 min. After washing, beads were incubated with RNase A and RNase H in DNA elution buffer. The eluted sample was then subjected to protease K treatment, DNA isolation and quantitative PCR analysis. Primers used for real-time PCR were listed in Supplemental Information Appendix, Table S1.

### Statistical analysis

Quantified results are presented as mean ± SEM. Comparisons between groups were evaluated using analysis of variance or the Student's *t-*test; *P* ≤ 0.05 was considered significant unless specified otherwise.

## DATA DEPOSITION

Gene expression data will be deposited within the Gene Expression Omnibus repository (www.ncbi.nlm.nih.gov/geo) under Neonatal Heart Maturation SuperSeries GSE85728 (http://www.ncbi.nlm.nih.gov/geo/query/acc.cgi?acc=GSE85728). All unique materials, resources, and reagents are available on request by qualified researchers for their own use. Generated plasmid constructs will be deposited in addgene.

## SUPPLEMENTAL MATERIAL

Supplemental material is available for this article.

## Supplementary Material

Supplemental Material
