# Supplementary material for: Ppp1r1b-lncRNA inhibits PRC2 at myogenic regulatory genes to promote cardiac and skeletal muscle development in mouse and human
Source: RNA. 2020 Apr;26(4):481–91. doi: 10.1261/rna.073692.119 (PMC7075267; doi:10.1261/rna.073692.119)
Supplement: Supplemental Material [file supp_26_4_481__index.html]

Ppp1r1b-lncRNA inhibits PRC2 at myogenic regulatory genes to promote cardiac and skeletal muscle development in mouse and human — Supplemental Material 

# Ppp1r1b-lncRNA inhibits PRC2 at myogenic regulatory genes to promote cardiac and skeletal muscle development in mouse and human

## Supplemental Material

- Supplemental\_Material.pdf
