# Supplementary material for: Ppp1r1b-lncRNA inhibits PRC2 at myogenic regulatory genes to promote cardiac and skeletal muscle development in mouse and human
Source: RNA. 2020 Apr;26(4):481–91. doi: 10.1261/rna.073692.119 (PMC7075267; doi:10.1261/rna.073692.119)
Supplement: Supplemental Material [file supp_073692.119_Supplemental_Material_.pdf]

Supplementary Information (SI)

Ppp1r1b-lncRNA Inhibits PRC2 at Myogenic Regulatory Genes to Promote Cardiac and Skeletal Muscle Development in Mouse and Human

Xuedong Kang[1,2], Yan Zhao[1,2], Glen Van Arsdell[3], Stanley F. Nelson[1.4.5,6], Marlin Touma*[1,2,5-9].

AUTHORS AFFILIATION

1. Department of Pediatrics, David Geffen School of Medicine, University of California Los Angeles, Los Angeles, CA.
2. Neonatal/Congenital Heart Laboratory, Cardiovascular Research Laboratory, University of California Los Angeles, Los Angeles, CA.
3. Department of Cardiothoracic Surgery, David Geffen School of Medicine, University of California Los Angeles, Los Angeles, CA.
4. Department of Neurology, David Geffen School of Medicine, University of California Los Angeles, Los Angeles, CA.
5. Department of Human Genetics, David Geffen School of Medicine, University of California Los Angeles, Los Angeles, CA.
6. Institute of Precision Health, David Geffen School of Medicine, University of California Los Angeles, Los Angeles, CA.
7. Children's Discovery and Innovation Institute, Department of Pediatrics, David Geffen School of Medicine, University of California Los Angeles, Los Angeles, CA.
8. The Molecular Biology Institute, David Geffen School of Medicine, University of California Los Angeles, Los Angeles, CA.
9. Eli and Edythe Stem Cell Institute, David Geffen School of Medicine, University of California Los Angeles, Los Angeles, CA.

***CORRESPONDENCE**

Marlin Touma, MD, PhD.
Department of Pediatrics, David Geffen School of Medicine, University of California, Los Angeles. 675 Charles E. Young Dr S, 3762 MacDonald Research Laboratories, Los Angeles, CA 90024. USA.
Tel: 310-825-6478 / Fax: 310-267-0154
Email: mtouma@mednet.ucla.edu

This PDF file includes:

Tables S1 to S4
Supplemental Figure 1, 2

Table S1. Primers used in real time PCR

	Sequence ((5' → 3')	Purpose
mPpp1r1b LncRNA	F: GCATCTGAGCAGCTGTGCAGCA	gene Expression; RIP
	R: CCTCCTCATCATCCTCCTGTGGGT	
mMyogenin	F: GCAGGCTCAAGAAAGTGAATGA	gene Expression
	R: TAGGCGCTCAATGTACTGGAT	
mMyoD1	F: CCACTCCGGGACATAGACTTG	gene Expression
	R: AAAAGCGCAGGTCTGGTGAG	
mDystrophin	F: GGAAAGCAACACATAGACAACCT	gene Expression
	R: GGGCATGAACTCTTGTAGATCC	
mTbx5	F: ACTTTGTGAGCACCCCAGCTGAGT	gene Expression
	R: CGATCTCAGTGTCCTGCTCTGCTCT	
hPpp1r1b LncRNA	F: AAGGCTGGGGACCTTCCAACTGAAG	gene Expression
	R: GGAGGAGAGTTCACTTCGTGAGCCAC	
hMyogenin	F: GGGGAAAACTACCTGCCTGTC	gene Expression
	R: AGGCGCTCGATGTACTGGAT	
hMyoD1	F: CGCCATCCGCTATATCGAGG	gene Expression
	R: CTGTAGTCCATCATGCCGTCG	
hDystrophin	F: AGCAAGAGCACAACAATTTGGT	gene Expression
	R: CCCTGTTCGTCCCGTATCATAA	
hTbx5	F: CTGTGGCTAAAATTCCACGAAGT	gene Expression
	R: GTGATCGTCGGCAGGTACAAT	
m h Troponin T2	F: CGTGAGGAGGAGGAGAACAG	gene Expression
	R: TCCTCTCTGCCAGGATCTTC	
mMyogenin promoter	F: GAGCCCCACTTCTATGATGG	CHIP; CHIRP
	R: GAAGAAAAGGGACTGGGGAC	
mMyoD1 promoter	F: GTCTCTCTGCCCTCCTTCCT	CHIP; CHIRP
	R: CAAGCTCCGCCCTACTACAC	
mTbx5 Promoter	F: TCCTTCCTTCCTTCCTTCCTCTTCC	CHIRP
	R: CCCATCCTCTTTACCCTCCTTCCAT	
hMyogenin promoter	F: AGCCTACCCTTCCTTGTTCC	CHIRP
	R: GAGGACACATTCCCCTCTCA	
hMyoD1 promoter	F: GCGTGTCTCTCAGCCTCTTT	CHIRP
	R: CTCCTCTGTCCCCTGATTTG	
hTbx5 Promoter	F: ACAAAATAGAGTGCCTCGTGCCTCG	CHIRP
	R: AATGCAATTCGTCCCAGAGTGGC	

Table S2. Antibodies

Antibody Name	Purpose	Manufacture
myosin, sarcomere	Immunofluorescence staining	Developmental Studies Hybridoma Bank, Iowa City, IA
Histone H3K27me3	CHIP	Active Motif, Carlsbad, CA
MyoD	Western	BD Biosciences, San Jose, CA
Myogenin	Western	NOVUS, Centennial, CO
Tropomyosin (Sarcomeric)	Western	Sigma, St. Louis, MO
Cardiac troponin T	Western	Abcam, Cambridge, MA
EZH2	Western, RIP	Cell Signalling Technology, Danvers, MA

Table S3. GapmeR and siRNA sequence

	Sequence ((5' → 3')	Target (Transcript ID)
GapmeR (mouse)	CGAGGAAAGGAACAGT	ENSMUST00000152525.1
siRNA (human)	CCCUCAUGGAUCCUUCUCUTT	ENST00000580029.1
	AGAGAAGGAUCCAUGAGGGTT	

Table S4. Capture probes used for ChIRP

Probe Name	Sequence ((5' → 3')
mPpp1r1b207-probe	TGCACAGCTGCTCAGATG/3BioTEG
hPpp1r1b206-probe mix	CTGGTAGAGAAGGATCCATGAG/3BioTEG
	CAAGCTAAGACTTAGTGCTGTG/3BioTEG
	AAGCTATCCAGGCAATAAAAGC/3BioTEG
	TCATAGATGCCAATCCACATAC/3BioTEG
	AGAGGAGACCGAGGAAAGCAAG/3BioTEG

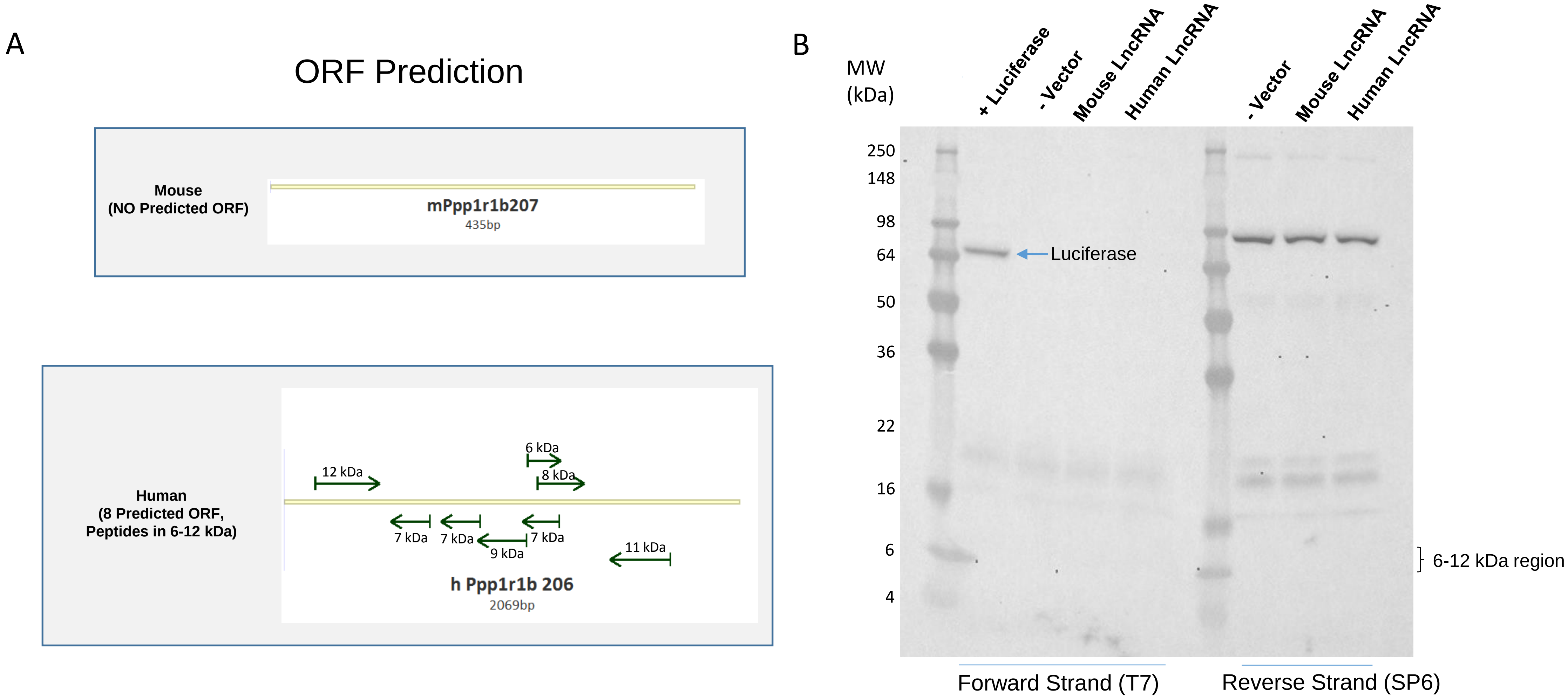

Supplemental Figure 1. *In vitro* transcription and translation assay. The full-length mouse and human lncRNA cDNA was cloned into pcmv sport 6.1 vector, which contains a T7 or a SP6 promoter. The Transend non-radioactive system was used to incorporated biotinylated lysine into nascent proteins. **A.** ORF prediction of Ppp1r1b-lncRNA in mouse and human orthologues. **B.** *in vitro* transcription-translation assay results. Right brace indicate the region of peptide bands on the western blot if they are expressed.

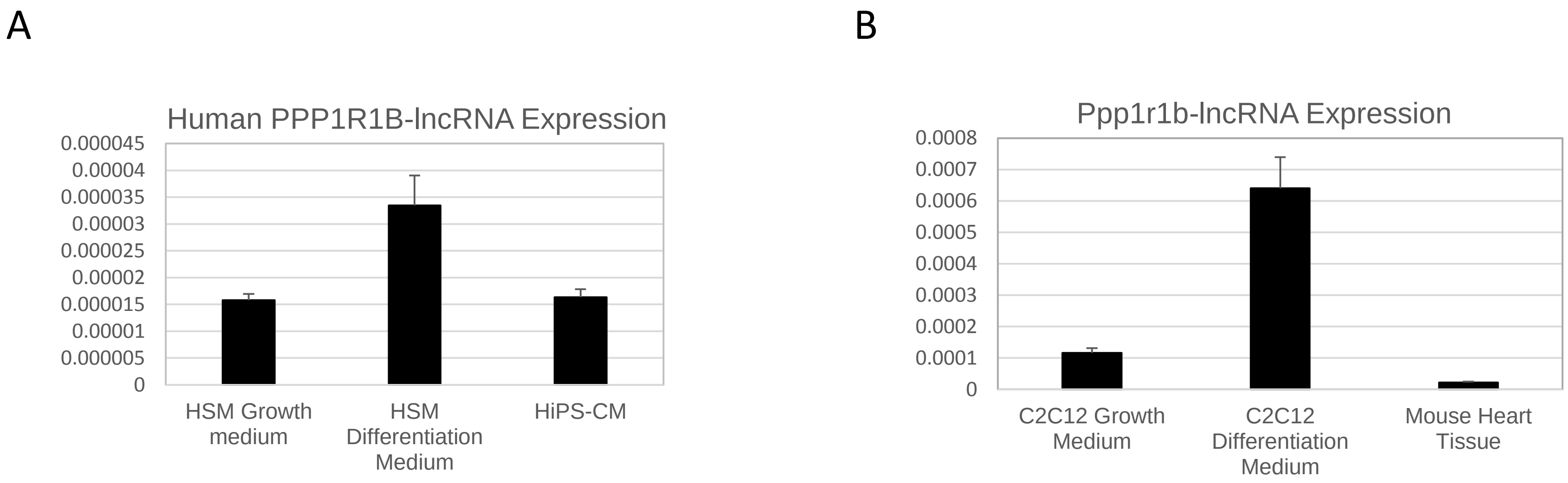

Supplemental Figure 2. *In vitro and in vivo* Expression of Ppp1r1b-lncRNA in our used systems. RT-PCR was performed with isolated RNA from different systems. **A.** Human PPP1R1B-lncRNA expression. Human skeletal myoblasts (HSM) before and after myotube differentiation as well as human iPSC-derived cardiomyocytes were used. **B.** Mouse Ppp1r1b-lncRNA expression. Mouse skeletal myoblasts (C2C12) before and after myotube differentiation as well as mouse neonatal heart tissue were used.
